# Phytochemicals Mediated Synthesis of AuNPs from *Citrullus colocynthis* and Their Characterization

**DOI:** 10.3390/molecules27041300

**Published:** 2022-02-15

**Authors:** Bismillah Mubeen, Mahvish Ghulam Rasool, Inam Ullah, Rabia Rasool, Syed Sarim Imam, Sultan Alshehri, Mohammed M. Ghoneim, Sami I. Alzarea, Muhammad Shahid Nadeem, Imran Kazmi

**Affiliations:** 1 Institute of Molecular Biology and Biotechnology, The University of Lahore, Lahore 54000, Pakistan; bismillah.mubeen@gmail.com (B.M.); mahvishrasool88@gmail.com (M.G.R.); inamgenetics@gmail.com (I.U.); rabia.amjad545499@gmail.com (R.R.); 2 Department of Pharmaceutics, College of Pharmacy, King Saud University, Riyadh 11451, Saudi Arabia; simam@ksu.edu.sa (S.S.I.); salshehri1@ksu.edu.sa (S.A.); 3 Department of Pharmacy Practice, College of Pharmacy, AlMaarefa University, Ad Diriyah 13713, Saudi Arabia; mghoneim@mcst.edu.sa; 4 Department of Pharmacology, College of Pharmacy, Jouf University, Sakaka 72341, Saudi Arabia; samisz@ju.edu.sa; 5 Department of Biochemistry, Faculty of Science, King Abdulaziz University, Jeddah 21589, Saudi Arabia

**Keywords:** green technology, AuNPs, *Citrullus colocynthis*, characterization, SEM, XRD, FTIR

## Abstract

Engineered nanoparticles that have distinctive targeted characteristics with high potency are modernistic technological innovations. In the modern era of research, nanotechnology has assumed critical importance due to its vast applications in all fields of science. Biologically synthesized nanoparticles using plants are an alternative to conventional methods. In the present study, *Citrullus colocynthis* (bitter apple) was used for the synthesis of gold nanoparticles (AuNPs). UV-Vis’s spectroscopy, XRD, SEM and FTIR were performed to confirm the formation of AuNPs. UV-Vis’s spectra showed a characteristic peak at the range of 531.5–541.5 nm. XRD peaks at 2 θ = 38°, 44°, 64° and 77°, corresponding to 111, 200, 220 and 311 planes, confirmed the crystalline nature of AuNPs. Spherical AuNPs ranged mostly between 7 and 33 nm, and were measured using SEM. The FTIR analysis confirmed the presence of phytochemicals on the surface of AuNPs. Successful synthesis of AuNPs by seed extract of *Citrullus colocynthis* (bitter apple) as a capping and reducing agent represents the novelty of the present study.

## 1. Introduction

**Figure 1:**
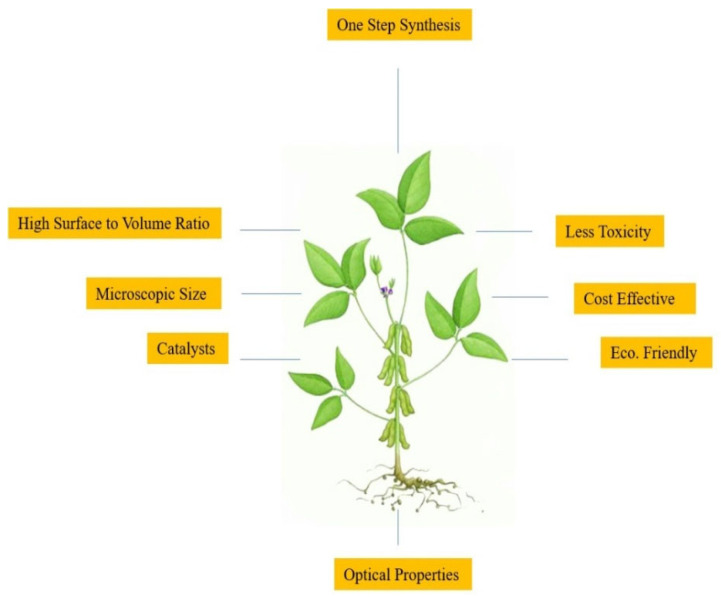
Important parameters of plant-mediated green technology.

Among modern technologies, nanotechnology, which deals with nanoscale materials ranging from 1 nm to 100 nm, is immensely popular due to its vast applications [1,2,3,4]. Professor Norio Taniguichi from Tokyo University of Science first introduced the term “nanotechnology” in 1974 [5]. Today, nanoparticles (NPs) are enlisted by experts in order to increase the yield and production, and their quality, in the manufacture of secondary metabolites, antioxidants, and as agents showing antimicrobial properties in plants [6,7,8]. Due to the novelty of engineered nanoparticles (ENPs), nanotechnology is being widely applied in all fields of medicine, such as in therapy for infections, allergies, diabetes, inflammatory issues and anti-cancer therapy [9,10,11,12]. Nanoparticles can also be used in textiles, food packing, household products, medical devices and in personal healthcare, due to their antifungal and antibacterial properties [13]. ENPs are also used for waste-water treatment [14]. In effect, this technology is becoming the need of the hour for many reasons, as it is most important for the production of device manufacturing at the level of gene or molecule. Further, this is marked as a multidisciplinary field of science, which has undergone numerous important developments [15]. Nanotechnology is also very important for the development and synthesis of new products for science, having a number of possible products and outcomes for the advancement of medicine. It is also helpful in the diagnosis of different diseases, for drug delivery and applications in bio-imaging and bio-sensing [16,17,18,19]. Nanotechnology is the scientific wonder of the modern era, having a broad spectrum and vast applications in numerous disciplines, especially in the domains of biology, physics and chemistry [20]. The term green nanotechnology is derived from green chemistry. The word “green” actually refers to the origin of substances from plants. Plants have been under consideration for many years, and now they have become the principal component in the manufacture of various nanoparticles [21]. Nanoparticles from plants are eco-friendly, risk-free, cost-effective, in a single one-pot protocol and time-efficient, and they have no high physical requirements for their formation. This is also associated with the vast applications of green engineering (Figure 1) [22,23].

There are numerous plants that could be used in the synthesis of nanoparticles [24]. Numerous researchers have been involved in the utilization of green synthesis and its different applications for various metal nanoparticles, as their need is increasing day by day due to their eco-friendly properties. Recently, numerous studies in this field have been in the limelight. Leaf, stem, fruit and seed-based extracts of plants are commonly used for eco-friendly synthesis of nanoparticles [25]. Green synthesis usually utilizes plant-based extracts as capping and reducing agents in the manufacture of nanoparticles, due to the reducing properties in the extracted leaves of plants [26]. The most basic concept about green technology is to synthesize new products that would definitely have a great potential for reuse. Such advanced technologies are basically used for advanced systems, in order to develop novelty or due to their providing a friendly environment for the production of new products [27].

**Figure 2:**
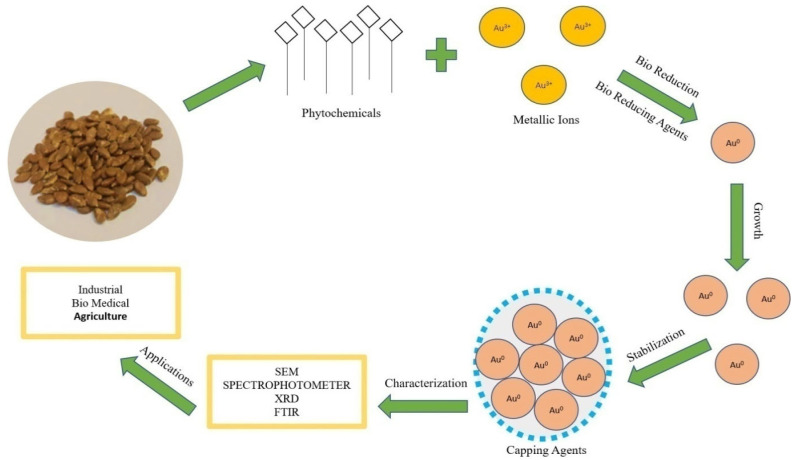
Mechanism of synthesis of Au-nanoparticles by using seed extract.

In vivo production of nanoparticles from plant extracts involves many biological resources [28]. The mechanism of nanoparticles synthesis (Figure 2) involves three main steps:

(1) Bioreduction of metallic ions to nanoparticles with the help of phytochemicals. Plant metabolites act as reducing agents [29]. The proposed mechanism of conversion (Figure 2) is Au^3+^ metallic ion to Au^0^ nanoparticles by plant bio reductants [30].

(2) Growth of nanoparticles; functional groups in plant extract (polyols and carboxylic acid) are responsible for the synthesis and growth of nanoparticles [31].

(3) Stabilization; secondary metabolites (secondary alcohols) are responsible for the bioreduction and stabilization of the nanoparticles. Nanoparticles synthesized by plants are more stable than other synthesis methods [32].

Biologically synthesized AuNPs (AuNPs) are found to be the most important choice or interest to experts and researchers because of their unique surface plasmon resonance (SPR), in contrast to other things [33]. AuNPs have been listed as a powerful nanoparticle for biomedical applications, due to their peerless abilities on the ground of optics [34]. AuNPs are biocompatible for application in many fields such as the food industry, pharmacology, medicine, water purification and others. AuNPs are also used in drug delivery, photothermal therapy, tumor imaging, sensing and labelling [35]. Green synthesis of AuNPs by plants such as tamarind [36], Aloe vera [37], *Cinnamomum camphora* [38] and *Salix alba* [39] have been reported. While numerous plants have been reportedly used in the synthesis of AuNPs, there is no evidence of synthesis of AuNPs from *Citrullus colocynthis* (bitter apple). *C. colocynthis* is an important member of the cucurbitaceous and is considered a plant of bitter flavor, with the seeds highly rich in oil and phytochemicals [40]. While this is a perennial plant with a long life cultivated as wild in sandy shores under the driest conditions, its fruits are found fleshy before maturity and turn dark green, commonly becoming yellow at maturity. However, these plants are of common medicinal origin from old times. The fruits of *C. colocynthis* were used in Asian and African countries generally for treating diabetics, microbial diseases, ulcer, various inflammations, jaundice and urinary diseases [41].

The present study focuses on in vivo eco-friendly biological synthesis of AuNPs from *Citrullus colocynthis* and their characterization using advanced analytical techniques such as UV-Vis spectroscopy, XRD, SEM and FTIR. In the reduction and synthesis of AuNPs, various biocompounds are involved. In this study, we used *C. colocynthis*. To the best of our knowledge no study has been done in context of AuNPs synthesis by *Citrullus colocynthis.* In a previous study it was reported that aqueous seed extracts of *C. Colocynthis* have chemicals compounds such as flavonoids that are responsible for antioxidant and other biological activities in plants [42,43]. In this simple biological synthesis these flavonoids act as bioreductants, reducing the gold atoms into gold ions, and are also responsible for stabilization of AuNPs.

## 2. Experimental Section

### 2.1. Materials

Seeds of *Citrullus colocynthis*, chloroauric acid, methanol, ethanol, HCl, HNO_3_, distilled water, Millipore water (Merck Millipore, Burlington, MA, USA), beakers, measuring cylinder, funnel, thermometer, pH meter, filter paper, water bath, Falcon tubes, Eppendorf tubes, centrifuge machine, lyophilizer and many others were used for analytical grades. The Millipore water used as solvent throughout the reaction and preparation procedures was obtained by filtering through Filter Tek with 0.45 µm pore size. All the glassware used in the synthesis process was thoroughly washed thrice with aqua regia (3 parts HCl and 1 part HNO_3_), rinsed with highly purified water and dried in the hot air oven.

### 2.2. Synthesis of Aunps

#### 2.2.1. Preparation of *Citrullus colocynthis* Seed Extract

Fresh seeds were collected from the fruit of *Citrullus colocynthis*. We ground 5 g seeds of *C. colocynthis* into powder. The seed extract was made into a 100 mL solution by adding distilled water and boiled for 10 to 15 min. After boiling, the extract was left for 2 h, filtered thrice to separate the crude and obtain the pure extract. This seed extract acted as a stabilizing and reducing agent to facilitate the synthesis of AuNPs.

#### 2.2.2. Preparation of Chloroauric Acid Stock Solution

Chloroauric acid (HAuCl_4_, H_2_O) was purchased from Burlington MA, USA. We prepared 1 mM of Chloroauric acid solution in 1000 mL reagent bottle in Millipore water and stirred for 10 min at room temperature. The solution was covered with aluminum foil and saved for further use.

#### 2.2.3. Synthesis and Purification of AuNPs

For the synthesis of AuNPs, aqueous seed extract was added to aqueous gold chloride solution at a fixed ratio of 1:2 (100 mL seed extract to 200 mL Chloroauric acid). This solution was placed in water bath for 20 to 30 min at the preadjusted temperature of 70 °C. Afterwards, active compounds of *C. colocynthis* and gold ions were allowed to react with each other. The color changed from yellowish to purple, showing the synthesis of AuNPs. The solution was allowed to cool at room temperature.

For the washing of AuNPs, the solution was centrifuged in HITACHI 1 High-Speed Refrigerated Centrifuge CR22N at 12,000 rpm for 1 h at 30 °C. After centrifugation, the AuNPs settled down in the form of pallets, which were collected, resuspended in Millipore water and centrifuged thrice at 12,000 rpm for 10 min, to remove undesirable particulates. After washing, the AuNPs were lyophilized using VaCO_2_ lyophilizer to obtain them in powder form.

### 2.3. Characterization of AuNPs

Synthesized AuNPs of *C. colocynthis* (bitter apple) were characterized by using various advanced analytical and instrumental techniques.

#### 2.3.1. UV-Vis Spectroscopy

UV-Vis’s spectrum was recorded by using a spectrophotometer (Model no. CECIL-7400S) to check the purity of AuNPs. For the preparation of sample for UV-Vis’s spectroscopy, 5 mg synthesized AuNPs were dissolved in 5 mL Millipore water and sonicated and vortexed alternately. When nanoparticles were completely segregated, 2 mL of gold nanoparticle solution was loaded in quartz cuvette. The UV-Visible spectrum was monitored by using the medium scan from 500–600 nm wavelengths and a step size of 10 nm per second.

#### 2.3.2. X-ray Diffraction (XRD)

X-ray diffraction analysis was conducted for structural information regarding synthesized AuNPs. A thin layer of well-ground powder form of AuNPs was placed on a glass slide, which was put in the XRD chamber. The chamber was closed and the X-ray diffraction meter, Philips PAN analytical X′pert Powder (WR14 1XZ Worcestershire, United Kingdom) adjusted. Using the Cu source at 2theta, the pattern of X-ray diffraction (XRD) was recorded with a scan range of 20° to 80°, step size of 0.02 with time per step of 20 to 30 s.

#### 2.3.3. Scanning Electron Microscope (SEM)

The powder form of synthesized AuNPs was used to obtain SEM images and to determine the shape and size of nanoparticles. The FEI Nova Nano SEM 450 system was used to analyze AuNPs. The experiment was conducted with a voltage of 5 KV. The gold nanoparticle sample was spread on carbon grid. The image was scanned at 10,000×, 25,000×, 50,000×, 100,000× and 200,000× magnification and photomicrographs were taken.

#### 2.3.4. FTIR Analysis

Fourier transform infrared spectroscopy (FTIR) helps in surface characterization of nanoparticles. To identify the surface reactive sites or functional groups to IR radiations responsible for surface reactivity, the pallets of synthesized AuNPs were placed in FTIR instrument (Cary 630 Agilent FTIR spectrometer) and the spectrum of AuNPs was recorded.

## 3. Results

**Figure 3:**
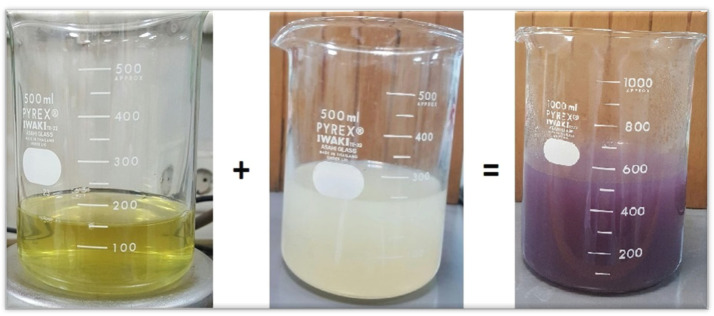
Yellow colored solution is Chloroauric acid, white colored solution is seed extract of *Citrullus colocynthis*, which when reacted then turned purple-colored, indicating the formation of AuNPs due to capping or reducing agents.

**Figure 4:**
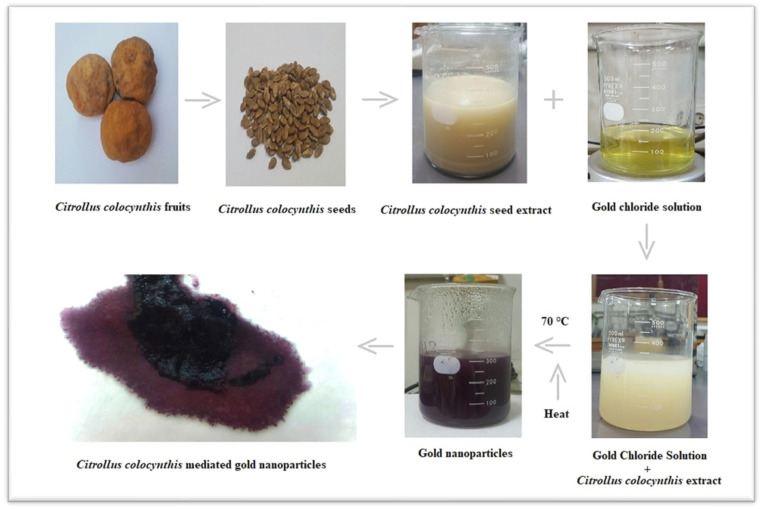
Flow chart shows synthesis of AuNPs from *C. colocynthis*.

In the synthesis method, the change in color from pale yellow to dark purple indicates the presence of gold atoms in the solution at nano scale. Au ions are reduced into Au atoms due to the reducing agent present in the seed extract of *Citrullus colocynthis*. In the reaction process, the color changed due to the formation of AuNPs and the surface plasmon response (SPR) in Figure 3 and Figure 4. This optical conformation has also been reported in literature [44].

### 3.1. UV-Vis Spectrophotometer Analysis

**Figure 5:**
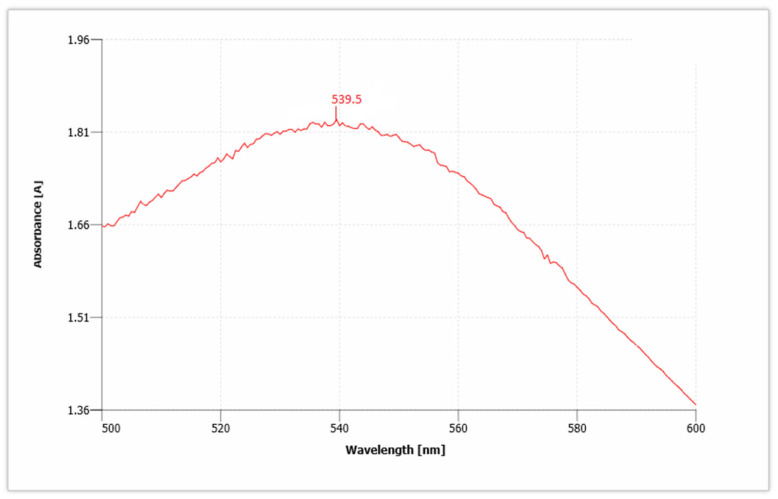
UV absorbance spectra show the presence of gold nanoparticles (AuNPs).

AuNPs have unique optical properties that are sensitive to shape and size, and also have a specific range of absorbance. Ultraviolet-Visible (UV-Vis) spectroscopy is used to check the SPR spectra of AuNPs. The UV absorbance spectra for synthesized AuNPs from *C. colocynthis* seed extract is shown in Figure 5. The wavelength for *Citrullus colocynthis* mediated AuNPs showed a maximum peak at 539.5 nm in accordance with the previous literature [45]. This confirms the presence of AuNPs, because AuNPs showed specific range of absorbance as compared to bulk that ranges between 500 nm and 600 nm. Our recent study is supported by previous findings [46,47]. Given that the spectra of AuNPs showed maximum surface plasmon response (SPR) between 531.5 and 543.5 nm that clearly showed different-sized nanoparticles are formed and broad peak of UV-Vis spectroscopy showed thickness in the gold deposited nanoparticles [48,49].

### 3.2. X-ray Diffraction (XRD) Measurements

**Figure 6:**
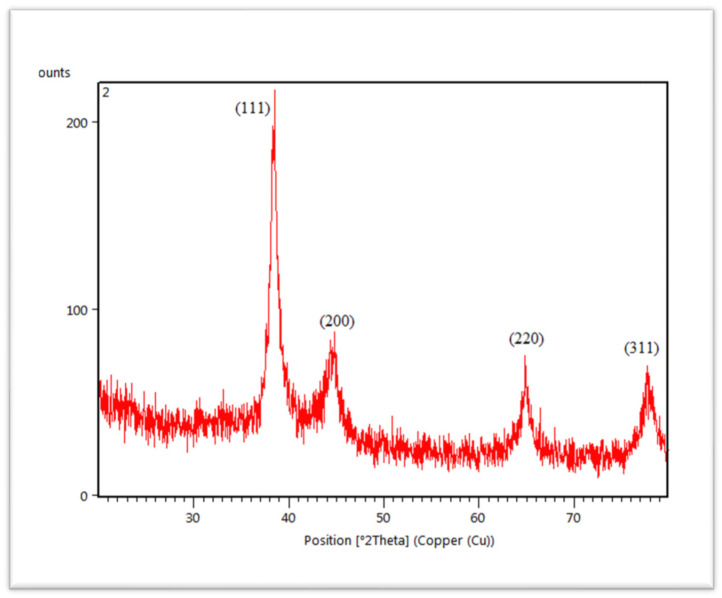
XRD spectra of *C. colocynthis*-mediated AuNPs.

The crystalline structure of synthesized AuNPs was characterized by X-ray diffractometer (XRD). The diffracted X-rays produced the characteristic X-ray diffraction patterns that determined the crystalline structure and the patterns are shown in Figure 6. AuNPs are present in the structure of nanocrystal. AuNPs showed four different diffraction peaks at about 2theta = 38°, 44°, 64° and 77°. According to standard Bragg reflections, all the four peaks corresponded to (111), (200), (220) and (311) plane, which shows face-centered cubic (FCC) structure of AuNPs. All four peaks in Figure 6 are sharp and narrow, suggesting a pure and well-crystallized structure of AuNPs. The most intense diffraction peak was observed at 2theta = 38°, which demonstrates the preferential growth along the (111) direction. This XRD pattern confirmed the presence of pure AuNPs [50].

### 3.3. Scanning Electron Microscope (SEM) Measurements

**Figure 7:**
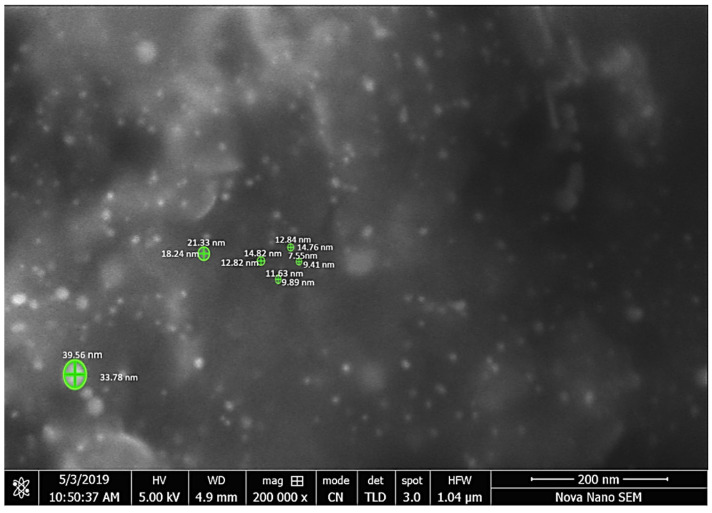
Microphotograph was scanned at 200,000× clearly showing the nanoparticles.

Further characterization was performed through scanning electron microscope. This is a simple and efficient method to determine the structural morphology, shape and size of the synthesized sample [51]. The structural morphology of *C. colocynthis*-capped AuNPs is shown in Figure 7. That indicates spherical shape particles with different size distributions. The size distribution according to SEM imaging is also mentioned in Table 1. The mean size of AuNPs was about 12 nm with sized distribution ranging from 7 nm to 33 nm.

Nanoparticles were measured with Fiji software used as image analyzer [52] (Table 1).

### 3.4. FTIR Analysis of AuNPs

**Figure 8:**
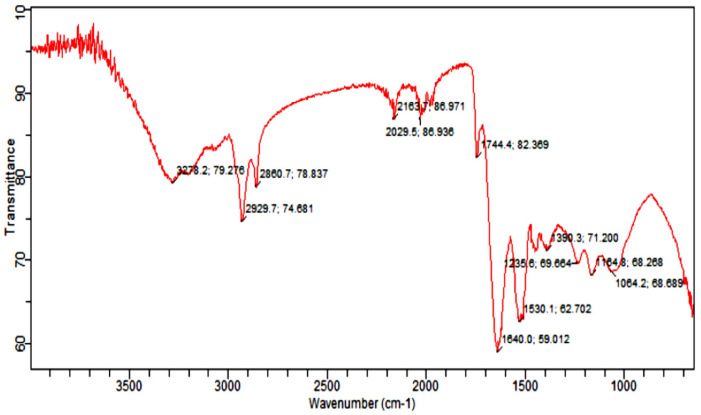
Infrared absorption bands of gold nanoparticle frequencies (cm^−1^).

Fourier Transform Infrared Spectroscopy Analysis (FTIR) was carried out to identify the functional group of active components, based on the peak value in the region of infrared radiation [53]. The results of FTIR peak values and functional groups are represented in Table 2, and the FTIR spectrum profile is illustrated in Figure 8, which represents strong bands of FTIR spectrum. The FTIR spectrum of *Citrullus colocynthis*-mediated AuNPs indicates the presence of various chemical groups’ residues that co-exist with the AuNPs. FTIR spectrum confirmed the presence of alcohol, phenol, alkenes, alkyl halide, amino acids, carboxylic acid, aromatic and amines.

FTIR analysis of the sample presenting the peak at 1640.0 cm^−1^ showed the presence of alkenes, while the peak at 2163.7 cm^−1^ marked the presence of (C≡C) stretch of alkynes compounds. Further, the presence of nitro (NO_2_ strech) was seen at 1390.3 cm^−1^, while the peaks at 1064.2 cm^−1^ and 1235.6 cm^−1^ marked the presence of Alkyl and Aryl halides C-F stretch. However, the peak at 3278.2 cm^−1^ marked the C-H compounds. The appearance of secondary metabolites on the surface of AuNPs such as flavonoids, phenols and amino acids has already been confirmed by literature [54,55].

## 4. Discussion

The present work deals with the synthesis of AuNPs, via *Citrullus colocynthis* seeds extract by a green synthetic system. Biologically synthesized nanoparticles were highly stable compared to synthetic nanoparticles due to capping proteins present in plant extracts. In the present study, seed extract is used as a green source and natural reductant for the formation and stabilization of metallic AuNPs [56]. The antioxidants present in the plant extract help the bioreduction of the gold solution. Addition of seed extract into Chloroauric acid solution led to the formation of purple color within 20 to 30 min. Gold ions present in solution reduced into gold atoms by bioreductants or biological capping agents that were present in seed extract. Initial purple color of the solution indicates presence of the AuNPs (Figure 3) and confirmed the reduction of Au ^3+^ into Au^0^. This optical conformation was also reported in literature [44]. Optical properties of resultant solution and change in color were highly dependent on size of nanoparticles. Dark color of resultant solution has larger nanoparticles [57].

On the other side, the size of biologically synthesized nanoparticles is highly dependent on plant extract. A higher amount of plant extract showed more production and forms the small-sized AuNPs, while decreasing the plant extract in gold solution gives the larger AuNPs with low yield [58]. The low concentration of *Corchorus olitorius* extract produced hexagonal and triangular shapes of AuNPs. A higher concentration of the extract produced quasispherical shapes of AuNPs [59]. In addition to plant extract concentration, gold (Au) salt concentration also affects the size of AuNPs. Mostly Chloroauric acid (HAuCl_4_) is used for green synthesis of AuNPs [60]. Adding a low concentration of gold solution into plant extract produced small size AuNPs. Instead of that, the addition of a high amount of gold chloride solution gradually increased the size of AuNPs [61]. To check the stability, shape, structure and size of the AuNPs, several techniques are done for their conformation. AuNPs absorb a specific wavelength of light with distinct optical features commonly referred to as surface Plasmon response (SPR). Usually, AuNPs give absorbance peaks between 500 nm and 600 nm. If a resultant sample showed a peak between 500 nm and 600 nm, that confirmed the presence of stabilized AuNPs. The present work confirmed the formation and stability of AuNPs with an absorbance peak at 539.5 nm in accordance with the previous literature [62,63]. The absorbance peaks of AuNPs are highly dependent on the size of the particles. Peaks at light wavelength indicated small-sized AuNPs. Broadness in peaks showed the presence of different-sized nanoparticles [64]. The absorption peak was found to be at 531 nm via aqueous leaf extract of *Lactuca indica* mediated AuNPs [65]. When HAuCl_4_ solution was treated with *Hibiscus sabdariffa,* the UV–Vis absorption spectra of AuNPs showed a characteristic peak at 532 nm [66].

Another technique used for characterization is XRD. The pattern of XRD demonstrates the peak intensity, shape, position and size of the nanoparticles that confirmed the purity and formation of synthesized nanoparticles. The XRD pattern of AuNPs (Figure 6) has also declared 4 diffraction peaks at 2theta = 38°, 44°, 64° and 77° are at (111), (200), (220) and (311) plane indexing face centered cubic (FCC) structure [67]. This revealed the crystalline nature of AuNPs [68,69].

The morphology and size of AuNPs were determined by using a scanning electron microscope. The SEM image was shown in Figure 7. AuNPs from *C. colocynthis* are spherical, and size ranged from 7 nm to 33 nm. Different-sized hexagonal AuNPs were also reported by a previous study [70]. AuNPs of 3–10 nm have already been reported from *Piper betle* leaf extract [71]. Furthermore, to estimate the presence of phytochemicals onto AuNPs, FTIR analysis was done. The FTIR spectra of AuNPs showed peaks at 3278 cm^−1^, 2929 cm^−1^, 2860 cm^−1^, 1744 cm^−1^, 1640 cm^−1^, 1390 cm^−1^,1235 cm^−1^, 1164 cm^−1^ and 1064 cm^−1^ as shown in Figure 8, which identify the possible biomolecules on the surface of synthesized AuNPs. The FTIR spectra clearly indicate the presence of water-soluble flavonoids, phenols and proteins present on the surface of AuNPs. These phytochemicals help in reduction and stabilization of nanoparticles [72,73].

## 5. Conclusions

In this study, biological synthesis of AuNPs was successfully carried out by using *C. colocynthis* seed extract that has not been used before for the synthesis of AuNPs. Seed extract was used as a green source and natural reductant for the formation and stabilization of AuNPs. The antioxidants present in the extract help the bioreduction of the gold solution. UV-Vis spectra, XRD, SEM and FTIR confirmed that biologically synthesized nanoparticles were highly stable due to the capping phytochemicals present in plant extracts, which protect the nanoparticles. The process of synthesis of AuNPs on a large scale from *C. colocynthis* is cost effective and biocompatible for several biological uses such as nanofertilizers, and bioherbicidal, biomedical and pharmaceutical applications. Further studies can be performed on *C. colocynthis* leaf extract, and Transmission electron microscopy (TEM) could be used for better images of nanoparticles.

## Figures and Tables

**Table 1:** Size of nanoparticles measured using Fiji.

	Area (nm^2^)	Mean	Perimeter (nm)	Feret Max (nm)	Feret Min (nm)
1	1017.622	112.943	122.13	39.56	33.784
2	126.917	36.705	44.323	14.819	12.823
3	131.026	40.439	45.442	14.757	12.838
4	277.118	66.565	66.369	21.335	18.243
5	48.85	89.065	27.219	9.411	7.554
6	81.264	45.719	33.812	11.625	9.886

**Table 2:** Infrared absorption bands of gold nanoparticle frequencies (cm^−1^).

Infrared Absorption Bands of AuNPs Frequencies (cm^−1^).							
S. No.	Esters Stretch C=O	Alkenes (C=C)	Alkynes (C ≡C) stretch	Alkane C-H	Nitro NO_2_ Strech	Alkyl & Aryl Halides C-F Stretch	Alcohol O-H Stretch
1	1744.4	1640.0	2163.7	2929.7 2860.7	1390.3	1064.2 1235.6	3278.2

## Data Availability

No data available.
